# Anticancer Effects of 1,3-Dihydroxy-2-Methylanthraquinone and the Ethyl Acetate Fraction of *Hedyotis Diffusa* Willd against HepG2 Carcinoma Cells Mediated *via* Apoptosis

**DOI:** 10.1371/journal.pone.0151502

**Published:** 2016-04-11

**Authors:** Yun-lan Li, Jiali Zhang, Dong Min, Zhou Hongyan, Niu Lin, Qing-shan Li

**Affiliations:** School of Pharmaceutical Science, Shanxi Medical University, Taiyuan 030001, PR China; Institute of Biochemistry and Biotechnology, TAIWAN

## Abstract

*Hedyotis Diffusa* Willd, used in Traditional Chinese Medicine, is a treatment for various diseases including cancer, owing to its mild effectiveness and low toxicity. The aim of this study was to identify the main anticancer components in *Hedyotis Diffusa* Willd, and explore mechanisms underlying their activity. *Hedyotis Diffusa* Willd was extracted and fractionated using ethyl acetate to obtain the H-Ethyl acetate fraction, which showed higher anticancer activity than the other fractions obtained against HepG2 cells with sulforhodamine B assays. The active component of the H-Ethyl acetate fraction was identified to be 1,3-dihydroxy-2-methylanthraquinone (DMQ) with much high inhibitory rate up to 48.9 ± 3.3% and selectivity rate up to 9.4 ± 4.5 folds (*p*<0.01) at 125 μmol/L. HepG2 cells treated with the fraction and DMQ visualized morphologically using light and fluorescence microscopy. Annexin V—fluorescein isothiocyanate / propidium iodide staining flow cytometry, DNA ladder and cell cycle distribution assays. Mechanistic studies showed up-regulation of caspase-3, -8, and -9 proteases activities (*p*<0.001), indicating involvement of mitochondrial apoptotic and death receptor pathways. Further studies revealed that reactive oxygen species in DMQ and the fraction treated HepG2 cells increased (*p*<0.01) while mitochondrial membrane potential reduced significantly (*p*<0.001) compared to the control by flow cytometry assays. Western blot analysis showed that Bax, p53, Fas, FasL, p21 and cytoplasmic cytochrome C were up-regulated (*p*<0.01), while Bcl-2, mitochondrial cytochrome C, cyclin E and CDK 2 were down-regulated dose-dependently (*p*<0.01). The reverse transcriptase-polymerase chain reaction showed that mRNA expressions of p53 and Bax increased (*p*<0.001) while that of Bcl-2 decreased (*p*<0.001). Pre-treatment with caspase-8 inhibitor Z-IETD-FMK, or caspase-9 inhibitor Z-LEHD-FMK, attenuated the growth-inhibitory and apoptosis-inducing effects of DMQ and the fraction on HepG2 cells. These results suggested that DMQ and the H-Ethyl acetate fraction of *Hedyotis Diffusa* Willd showed potential anticancer effects. Furthermore, the mechanisms of action may involve mitochondrial apoptotic and death receptor pathways.

## Introduction

Cancer is a type of refractory disease with high morbidity and mortality, which has raised attention worldwide, for the search of novel and effective therapeutic approaches. Traditional chemotherapy and radiotherapy have intrinsic and potential cytotoxic effects on normal cells. This cytotoxicity coupled with severe toxicity and adverse side effects, such as hair loss, vomiting, and nausea, limit their long-term application. The limitations of these therapies highlight the urgent need to find safer and more selective treatment options with fewer side effects for cancer therapy. *Hedyotis Diffusa* Willd (HDW) belongs to the Rubiaceae family and is a natural medicinal herb widely distributed in China. It has long been used as an important component in several chinese medicine formulations for the treatment of inflammatory diseases such as appendicitis, urethritis and bronchitis. It has also been used for various kinds of cancers including liver, lung, colon and thymus, with relatively fewer and milder side effects [1].

The definition of apoptosis was first introduced by Kerr et al., in 1972 to distinguish it from necrotic cell death [2]. Apoptosis is an adenosine triphosphate (ATP) -dependent programmed physiological cell suicide, which is essential for the elimination of excess, redundant, and abnormal cells to maintain tissue homeostasis. Inhibiting the excessive proliferation or promoting apoptosis of tumor cells is also one of the key approaches for the development of anticancer drugs. The cysteine aspartic acid-containing proteases (caspase) family can be categorized into initiators (such as caspase-8 and 9) and effectors (such as caspase-3). The caspases are activated by cleavage, which further activates the downstream substrate molecules [3]. Caspase-9 and 8 are usually involved in the mitochondrial apoptotic and death receptor pathways, respectively, while caspase-3 is involved in both pathways. P53, a proapoptotic factor acts on proteins (such as Fas, Bax, Bcl-2, and p21) to participate in several signal pathways including the mitochondrial apoptotic pathway, death receptor pathway, and cell cycle arrest [4, 5]. The Bcl-2 family of proteins is classified according to the structure of their short conserved Bcl-2 homology domains and a C-terminal tail. The Bcl-2 family includes Bax and Bcl-2, which are involved in the mitochondrial apoptotic pathway [6–8]. Extracellular protein death ligands of the tumor necrosis factor (TNF) family bind to receptors such as FasL, which interacts with Fas and activates the death receptor pathway. In addition, CDK 2 and cyclin E are the main cell cycle proteins involved in the G0/G1 stage of the cell cycle [9].

The compound DMQ, also called as rubiadin, was first isolated and purified from HDW [10], which possesses diverse pharmacological effects such as potent anticancer activity *in vitro* [11], and hepatoprotection against carbon tetrachloride (CCl_4_) -induced damage in rats [12]. In addition, it has an antioxidant property, which was found to be more effective than ethylenediaminetetraacetic acid (EDTA), Tris, mannitol, vitamin E, and p-benzoquinone [13] and photodynamic activity on cancer cells [14, 15]. Despite reports of the anticancer activity of DMQ, the precise mechanism and signal pathways involved were not reported in the literature. In the present study, the ethanol extract of HDW was fractioned with ethyl acetate, and the components were analyzed for anticancer activity. The HDW ethyl acetate (H-EtOAc) fraction and DMQ were selected for further elucidation of their exact anticancer mechanisms. To the best of our knowledge, this is the first study of the mechanisms of apoptosis and cell cycle arrest of HepG2 carcinoma cells *in vitro*, induced by DMQ and the H-EtOAc fraction.

## Materials and Methods

### Plant Materials and Reagents

*Hedyotis diffusa* Willd was purchased from Guilin (Guangxi, RP China) and authenticated by Professor Tianai Gao from Shanxi Institute for Food and Drug control. Fetal bovine serum (FBS), RPMI-1640, penicillinG, streptomycin, trypsin, bicinchoninic acid assay (BCA) and the enhanced chemiluminescence (ECL) kits were obtained from Wuhan Boster Biological Engineering Co., Ltd. (Wuhan, PR China). Sulphorhodamine B (SRB) powder was purchased from J&K Scientific Ltd. (Beijing, RP China). Acridine orange (AO) and ethidium bromide (EB) were obtained from Amerisco (Solon, Ohio, USA). AnnexinV-fluorescein isothiocyanate/propidine iodide (Annexin V-FITC/PI) apoptosis, DNA ladder and cell cycle detection kits were purchased from KeyGen technology (Nanjing, RP China). Mitochondrial membrane potential (MMP) detection kit was obtained from the Beyotime Institute of Biotechnology (Shanghai, RP China). Reactive oxygen species (ROS) and caspase-3, -8 and -9 activity assay kits were purchased from Applygen Technologies, Inc. (Beijing, PR China). Cytoplasmic and mitochondrial protein extraction kit, antibodies against p53, Bax and primers of p53, Bax, Bcl-2 and β-actin were obtained from Shanghai Sangon Biological Engineering Technology and Service Co., Ltd. (Shanghai, PR China). Antibodies against cyclin E, CDK 2, Bcl-2, p21 and cytochrome C (cyto C) were purchased from Cell Signaling technology (Beverly, MA, USA). Inhibitors of caspase-8 (Z-IETD-FMK), caspase-9 (Z-LEHD-FMK) and antibodies against Fas and FasL were purchased Santa Cruz Biotechnology, Inc. (Santa Cruz, CA). P53 inhibitor pifithrin-α (PFT-α) was purchased from Sigma-Aldrich (Sigma Aldrich, MO, USA) Anti-rabbit or anti-mouse secondary antibodies were purchased from Beijing Zhong Shan-Golden Bridge Biological Technology Co., Ltd. (Beijing, RP China). The RNA extraction kit TRNzol, FastQuant RT Kit (with gDNase) and 2 × Taq PCR MasterMix were obtained from TianGen Biotech Co., Ltd. (Beijing, PR China). GelRed^™^ Nucleic Acid Gel Stain was obtained from Biotium, Inc. (California, USA). PageRuler Prestained Protein ladder and GeneRuler 100bp DNA Ladder was purchased from Thermo Fisher Scientific Inc. (Shanghai, PR China).

### Sample Extraction

HDW segments were successively extracted (H-Total extract) and dissolved in water, then fractionated with ethyl acetate (EtOAc) and *n*-butanol (*n*-BuOH) to obtain H-H_2_O, H-EtOAc and H-*n*-BuOH fractions. Six components, including rutin, quercetin, 6,7-dihydroxycoumarin, DMQ, geniposidic acid and methyl hexadecanoate in H-EtOAc fraction, were isolated by column chromatography on silica gel in our experimental group. The HPLC-Q-Orbitrap method was applied to identify the composition of ethyl acetate extract of *Hedyotis diffusa*. Ultimate 3000 RSLCnano high performance liquid chromatograph (Thermo Fisher Scientific, MA) with hyper Gold C18 chromatographic column (100 mm × 2.1 mm, 1.9 μm) was used for separation of compounds. The mobile phase for HPLC analysis consisted of 0.1% formic acid in water (solvent A) / acetonitrile (solvent B) with a flow rate of 0.3mL/min. High-resolution mass spectrometer Q-Orbitrap HRMS (Thermo Fisher Scientific, MA) with HESI-II ESI ion source were used for identification of six compounds. With the help of XCalibur and Mass Frontier 7.0 software, we speculated the possible fragmentation pathway of every composition, matching them with the database in Mass Frontier.

### Cell Lines and Cell Culture

The following human cell lines (their gene backgrounds had been identified by the company) were employed in the current study, hepatocellular carcinomas (HepG2), embryonal carcinoma (EC), cervical carcinoma (SiHa), breast carcinoma cell (MCF-7), gastric carcinoma (SGC-7901), lung carcinoma (A549) and normal hepatocyte cell line (HL-7702) were obtained from Wuhan boster Biological Engineering Co., Ltd (Wuhan,PR China) and incubated in RPMI-1640 medium supplemented with 10% fetal bovine serum, 1% penicillin/streptomycin, 5% CO_2_ at 37°C humidified atmosphere. The cell lines were purchased from Wuhan Boster Biological Engineering Co., Ltd. (Wuhan, PR China). All cells used in the experiments were in logarithmic growth phase.

### Cytotoxicity

Cell inhibitory rates were quantified by SRB assay to compare the effects of different fractions. Cells were seeded at a density of 2×10^5^ cells per well in 96-well plates. After attachment, in the present or absent of Z-IETD-FMK (20 μmol/L) or Z-LEHD-FMK (20 μmol/L) for 30 min, the cells were treated with different extracts from HDW (H-Total, H-EtOAc, H-*n*-BuOH and H-H_2_O fractions) at 62.5, 125, 250 and 500 μg/mL or DMQ at 62.5, 125, 250 and 500 μmol/L for 24 h. Cells were fixed for 1 h at 4°C by adding 50 μL of 30% (w/v) trichloroacetic acid (TCA). Then, the supernatant was discarded and the wells were washed with deionized water until the TCA was almost cleaned and air dried. The cells were incubated in 100 μL of 0.4% (w/v) SRB dissolved in 1% acetic acid at room temperature for 20 min, washed gently with 1% acetic acid to remove the uncombined SRB colorant, air dried and dissolved in 150 μL of 10 mmol/L tris-base solution (pH 10.5). Then the plates were agitated for 30 sec and the absorbance values were read at 515 nm using a microplate reader (Thermo Fisher scientific Inc.). Cell inhibitory rates were calculated as the following formula: Inhibitory rates % = (absorbance of untreated cells—absorbance of treated cells) / absorbance of untreated cells × 100%.

SRB assay was also performed to compare the activities of six components from H-EtOAc fraction on cells. HepG2 and HL-7702 cells were seeded at a density of 2×10^5^ cells per well in 96-well plates and attached, in the present or absent of Z-IETD-FMK (20 μmol/L), Z-LEHD-FMK (20 μmol/L) for 30 min, 100 μL of rutin, quercetin, 6,7-dihydroxycoumarin, DMQ, geniposidic acid and methyl hexadecanoate with gradient concentrations (5, 50, 125, 250 and 500 μmol/L) were added and cultured for 24 h. The following steps were operated in the same way as above. The calculation of selectivity rate was: Selectivity = inhibitory rates on HepG2 cells / inhibitory rates on HL-7702 cells.

### Cell Morphology Changes

When HepG2 cells at a density of 2×10^4^ cells per well in 96-well plates were treated with H-EtOAc fraction (0, 125 and 250 μg/mL) and DMQ (197, 492 and 983 μmol/L) for 24 h respectively, cell morphology was observed and photographed under light microscope 20 × magnification using a visible light (BDS200-PH Inverted Microscope, Chongqing Optec Instrument Co., Ltd., China).

### AO/EB Fluorescence Staining

AO can pass through cell membrane and embed into DNA, which presents green fluorescence. EB can only transcend disruptive cell membrane, which binds with DNA and sends out orange fluorescence. AO coupled with EB is a classical way to observe apoptosis. Cell nucleus showed green normal morphology in the control group, while yellow or orange cell nucleus was displayed in apoptosis cells. The quantification of apoptotic cells was calculated according to Ribble [16], where apoptotic cells showed yellow or orange condensed or fragmented chromatin. At least 600 nuclei per pellet were scored using a fluorescence microscope at a magnification of 40×and apoptotic cells were determined [17] and the percentage of apoptotic cells within the overall population of total cells was defined as Apoptosis rate.

HepG2 cells were plated in 24-well plates at a density of 2×10^4^ cells per well. After attachment, cells were treated with DMQ (0, 20, 200 and 500 μmol/L) and H-EtOAc fraction (0, 125 and 250 μg/mL) for 24 h respectively, then stained with dye mixture containing 100 mg/L AO and 100 mg/L EB solution of each for 5 min according to the protocol and examined by fluorescence microscope using a green excitation wavelength (460–495 nm) and emission wavelength 510 nm filter under 10 × or 40 × magnification (Olympus, IX-53, Japan).

### AnnexinV-FITC/PI Staining Flow Cytometry Assays

The externalization of phosphatidylserine (PS) to outer plasma membrane is a characteristic marker for early apoptosis. AnnexinV is a Ca^2+^-dependent phospholipid-binding protein, which can not transcend cell membrane, but it binds to PS with high affinity. We can detect the green fluorescence by tabbing annexin V with FITC fluorescein. Besides, PI, a nuclei dye, can send out red fluorescence when combined with cell nucleus in late apoptosis and dead cells. So, Annexin V-FITC accompanied with PI can distinguish living cells, early apoptosis cells, late apoptosis and dead cells.

To detect the apoptosis rates, HepG2 cells were incubated in 6-well plates at a density of 2×10^5^ cells per well. After treatment with DMQ (0, 79 μmol/L for 12 h, 157 μmol/L for 12 h and 79 μmol/L for 24 h) and H-EtOAc fraction (100 μg/mL for 12 h, 400 μg/mL for 12 h and 100 μg/mL for 24 h), or in the present of Z-IETD-FMK (20 μmol/L), Z-LEHD-FMK (20 μmol/L) for 30 min, cells were collected, washed and resuspended in 500 μL binding buffer according to the manufacturer’s protocol. The mixture of 5 μL Annexin V-FITC with 5 μL PI were added and reacted in the dark for 15 min at room temperature. The samples were injected into the flow cytometer (Accuria C6, BD, America) within 1 h. The cells were counted at least 5×10^5^ and only single cells were considered. Fluorescence excitation wavelength was set at 488 nm, and emission at 530 nm. Fluorescence signal of FITC was detected at FL 1 passage, and PI at FL 2 passage. Electronic compensation was conducted and the data was analyzed.

### DNA ladder assays

HepG2 cells (3×10^6^) collected after treatment with DMQ (0, 79 μmol/L for 24 h, 157 μmol/L for 12 h and 157 μmol/L for 24 h) and H-EtOAc fraction (400 μg/mL for 24 h) were washed with PBS and resuspended in 20 μL lysis buffer gently. 10 μL Enzyme A was added to incubate 2 h at 37°C, and incubation was continued at 50°C for further 2 h after 10 μL Enzyme B was added. Fragment DNA was separated on 1.5% agarose gel for 4 h at 50 V and then stained with GelRed and photographed.

### Caspase-3, -8 and -9 Proteases Activities

Caspase-3, -8 and -9 proteases activities were determined according to the manufacturer’s instructions of caspase-3, -8 and -9 colorimetric assay kits which were purchased from Applygen Technologies, Inc. (Beijing, PR China) for caspase activity evaluation. Briefly, DMQ (0, 79 μmol/L for 12 h, 157 μmol/L for 12 h and 79 μmol/L for 24 h) and the H-EtOAc fraction (100 μg/mL for 12 h, 400 μg/mL for 12 h and 100 μg/mL for 24 h) treated HepG2 cells (1×10^6^) were resuspended in 100 μL of cell lysis buffer, incubate on ice for 15 min, centrifuged for 15 min at 18000 g, then the supernatants (lysised protein) were transferred to a chilled fresh tube and put on ice for immediate assay or stored at -80°C for future use. Protein was measured by the Bradford method, as described in the Bio-Rad protein assay kits and adjusted to a concentration of 1–3 μg/μL. Reaction buffer was added to 30 μL protein samples to a consistent volume, and 5 μL of the 10 mmol/L Ac-DEVD-pNA (caspase-3) / Ac-IETD-pNA (caspase-8) / Ac-LEHD-pNA (caspase-9) was added and incubated at 37°C for 2 h. Samples were readed at 405 nm in a microplate reader (Thermo Fisher scientific Inc.).

### Mitochondrial Membrane Potential Assay

5,5',6,6'-tetrachloro-1,1',3,3'-tetrethyl benzimidazoly carbocyanine iodide dye (JC-1) is an ideal fluorescent probe, which is widely used to detect mitochondrial membrane potential (MMP). Aggregated JC-1 in matrix can transmit red fluorescence (emission at 585 nm, excitation at 590 nm) in high MMP cells, while green fluorescence (emission at 514 nm, excitation at 529 nm) in low MMP cells, as JC-1 can not gather together, being monomers in mitochondrial matrix. We can easily detect the transition from red fluorescence to green, and the ratio between red and green fluorescence is an index of MMP, a hallmark of early apoptosis related to mitochondrial apoptotic pathway.

The decline of MMP was measured by JC-1 according to the instruction using a flow cytometer (Accuria C6, BD, America). After exposed to DMQ (0, 79 μmol/L for 12 h, 157 μmol/L for 12 h and 79 μmol/L for 24 h) and H-EtOAc fraction (100 μg/mL for 12 h, 400 μg/mL for 12 h and 100 μg/mL for 24 h) in a 6-well plate, HepG2 cells (2×10^5^) were harvested, washed with PBS twice and incubated with JC-1 dye for 20 min at 37°C. In the following, the cells was centrifuged, washed and resuspended with JC-1 buffer solution before measurement. Fluorescence signal of red was detected at FL 2 passage, and green at FL 1 passage. The changes of the ratio between red and green fluorescence represent the alternation of MMP.

### ROS Assay

20,70-dihydrofluorescein-diacetate (DCFH-DA) can cross cell membrane freely and be hydrolyzed into 20,70-dihydrofluorescein (DCFH), which can be oxidized by ROS into dichlorofluorescein (DCF). The fluorescent intensity of DCF can reflect the ROS levels in cells. The intracellular ROS promotion was detected by DCFH-DA. HepG2 cells (2×10^5^) in 6-well plate were treated with DMQ (0, 79 μmol/L for 12 h, 157 μmol/L for 12 h and 79 μmol/L for 24 h) and H-EtOAc fraction (100 μg/mL for 12 h, 400 μg/mL for 12 h and 100 μg/mL for 24 h) and following 10 μmol/L DCFH-DA for another 60 min in the incubator. Then cells were harvested, washed and resuspended in 500 μL PBS before measurement by the flow cytometer (Accuria C6, BD, America) at a fluorescence excitation wavelength of 488 nm and emission at 525 nm. Fluorescence signal of DCFH-DA was detected at FL 1 passage. The cells were counted at least 5×10^4^ and only single cells were considered.

### Cell Cycle Distributions by Flow Cytometry Analysis

To detect the effects on cell cycle, HepG2 cells (2×10^5^) were seeded in 6-well plates. After attachment, HepG2 cells were treated with DMQ (0, 79 μmol/L for 12 h, 157 μmol/L for 12 h and 79 μmol/L for 24 h) and H-EtOAc fraction (100 μg/mL for 12 h, 400 μg/mL for 12 h and 100 μg/mL for 24 h). Then the cells were harvested, washed with PBS for 3 times and fixed overnight at 4°C in 70% ethanol. Before measurement on the flow cytometer (Accuria C6, BD, America), 100 μL of RNase was added and incubated for 30 min at 37°C, and then 400 μL PI dye for 30 min at 4°C. Excitation wavelength was set at 488 nm and PI fluorescence signal was detected at FL 2 passage. At least 5×10^5^ cells were monitored and only single cells were considered. The cell cycle distributions were analyzed by Modfit software (Verity Software House, USA).

### Western Blot Analysis

After DMQ (0, 79, 157 and 315 μmol/L) and H-EtOAc fraction (250 μg/mL) treatment for 24 h, HepG2 cells (1×10^7^) were harvested, lysed in a lysis buffer containing 1% phenylmethanesulfonyl fluoride (PMSF) for 3 times of freezing-thawing and centrifuged at 12000 rpm for 15 min at 4°C. The protein concentration of the supernatant was determined by BCA assay. About 50 μg proteins from different groups and 3 μL marker were loaded in each lane, separated on the 12% sodium dodecyl sulphate-polyacrylamide gels (SDS-PAGE) and followed by electrophoretically transfer of the proteins to nitrocellulose (NC) membrane. Then the NC membrane was blocked with 5% (w/v) nonfat dry milk dissolved in tris-buffered saline plus Tween-20 (TBST) solution for 2 h and reacted with relevant apoptosis antibodies (p53, Bax, Bcl-2, cyto C, Fas L, Fas, p21, cyclin E and CDK 2) for 20 h at 4°C, in which β-actin was used as the internal control, followed by incubated with peroxidase-conjugated secondary antibodies. Membranes were detected using enhanced ECL detection kits. The strength of protein expression signal were analyzed by scanning densitometry using a Microtek ScanMaker 8700 (Zhongjing Inc. China) with ScanWizard 5 software.

### RT-PCR Analysis

HepG2 cells (1×10^5^) treated with DMQ (0, 79 μmol/L for 12 h, 157 μmol/L for 12 h and 79 μmol/L for 24 h) and H-EtOAc fraction (100 μg/mL for 24 h) were lysised by 1 mL of TRNzol, and mRNA was extracted according to the manufacturer’s instructions. Concentration and purity of mRNA were calculated according to the absorbance at 260 nm and 280 nm spectrophotometrically. DNA was detached and the total mRNA was reverse transcripted into cDNA at 42°C for 15 min and 95°C for 3 min using the FastQuant RT Kit (with gDNase). Table 1 shows the synthetic oligonucleotide primers used for RT-PCR and the product sizes. Single stranded cDNA was used as a template for PCR amplification using Taq polymerase. The amplification was performed by 2 × Taq PCR MasterMix under the following conditions: predegeneration at 94°C for 3 min for 1 cycle, degeneration at 94°C for 30 sec, annealing at (Tm-5)°C for 30 sec, then extension at 72°C for 1 min for 30 cycles, 72°C for 5 min for 1 cycle at late extention. In the following, 5 μL sample aliquots were electrophoresed on a 1.5% (w/v) agarose gel for 50 min at 120 V and then stained with GelRed and photographed. The strength of mRNA expression signal were analyzed by scanning densitometry using a Microtek ScanMaker 8700 (Zhongjing Inc. China) with ScanWizard 5 software.

**Table 1:** Primers Used for RT-PCR.

Genes	Forward primer (5’-3’)	Reverse primer (5’-3’)	Product size (bp)
β-actin	CTACAATGAGCTGCGTGTGGC	CAGGTCCAGACGCAGGATGGC	270
Bax	TGCTTCAGGGTTTCATCCAGGA	ACGGCGGCAATCATCCTCTG	172
Bcl-2	CTTCGCCGAGATGTCCAGCCA	CGCTCTCCACACACATGACCC	152
p53	CTGGCCCCTGTCATCTTCTG	CCGTCATGTGCTCCGCTAACC	242

### Statistical Analysis

Average values with their standard deviation were presented (mean ± SD). * (*p*<0.05), ** (*p*<0.01) and *** (*p*<0.001) indicated significant differences compared to the control, which were calculated by two-tails t-test.

## Results

### The Fraction and Component which Owned Higher Anticancer Viability and Selectivity from Hedyotis Diffusa Willd

**Fig 1:**
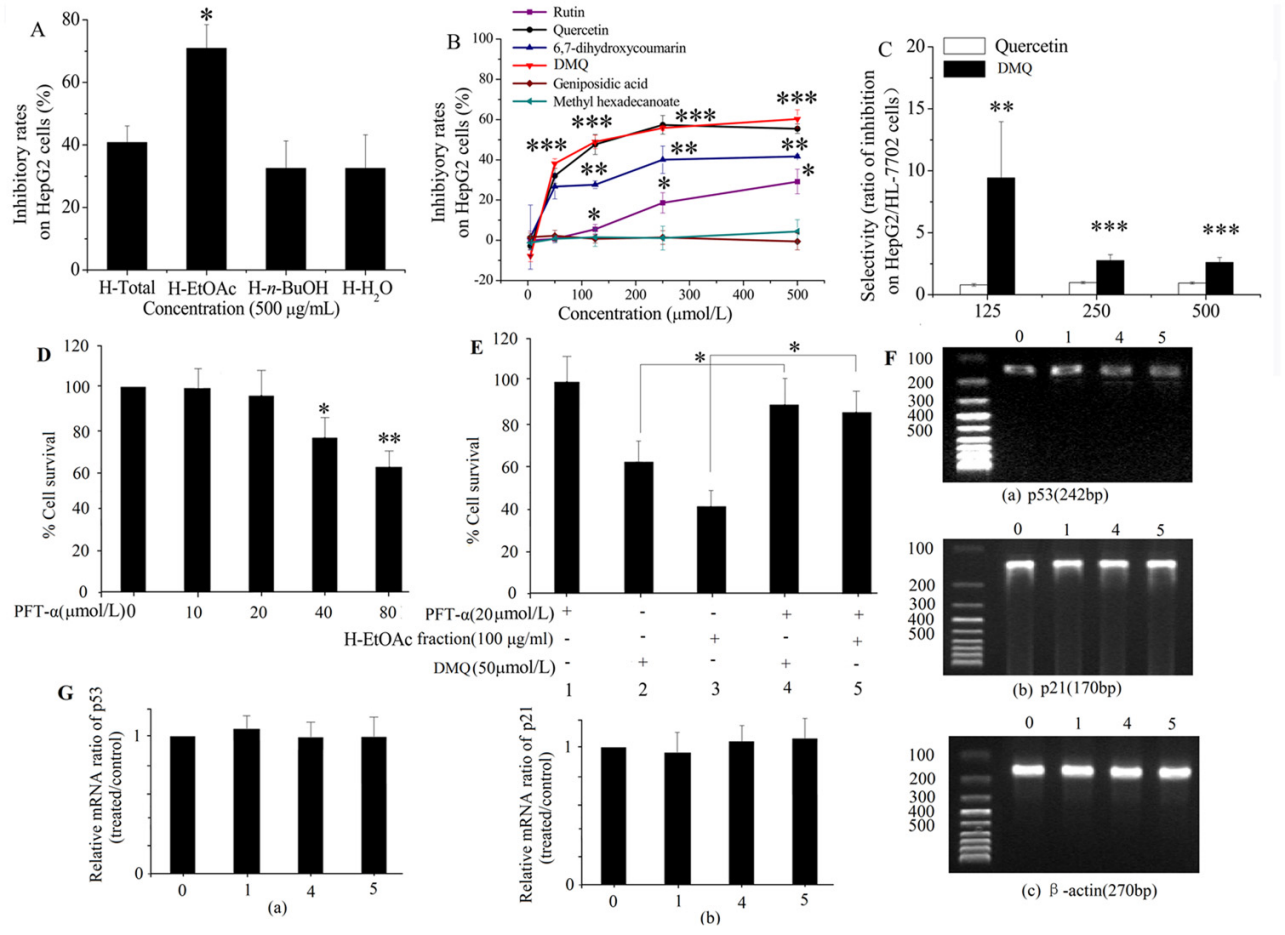
The Anticancer Effects of H-EtOAc Fraction and DMQ. A. The inhibitory effects of four fractions on HepG2 cells at 500 μg/mL for 24 h. B. The inhibitory effects of six indentified components on HepG2 cells at gradient concentrations (0, 5, 50, 125, 250 and 500 μmol/L) for 24 h (the inhibitory effect of geniposidic acid group was the control). C. The selectivity rates of two high anticancer components between HepG2 and HL-7702 cells (the selectivity of quercetin was the control). D. HepG2 cells were treated with varying concentrations of PFT-α (0–80 μmol/L) for 24 h and cell survival was evaluated by SRB assay (0 μmol/L PFT-α group was the control). E. Viability of HepG2 cells were treated with/without indicated concentration of DMQ and H-EtOAc fraction plus PFT-alpha for 24 h by SRB assay (the group of 20 μmol/L PFT-α without DMQ and H-EtOAc fraction treatment was the control). F. RT-PCR results of p53, p21and β-actin in HepG2 cells. G. mRNA levels of p53 and p21 in HepG2 cells. Line 0: the control (without PFT-α, DMQ and H-EtOAc fraction). Line 1: PFT-α (20 μmol/L) without DMQ and H-EtOAc fraction. Line 2: DMQ (50μmol/L) and PFT-α (0 μmol/L). Line 3: H-EtOAc fraction (100 μg/ml) and PFT-α (0 μmol/L) Line 4: DMQ (50μmol/L) and PFT-α (20 μmol/L). Line 5: H-EtOAc fraction (100 μg/ml) and PFT-α (20 μmol/L). The strength ofmRNA expression signal were analyzed by scanning densitometry using a Microtek ScanMaker 8700 (Zhongjing Inc. China) with ScanWizard 5 software. Values are means ± SD of three independent experiments. * (*p*<0.05), ** (*p*<0.01) and *** (*p*<0.001) represented significant differences compared to the control.

H-EtOAc fraction exerted the higher inhibitory rates up to 71.2 ± 7.5% at 500 μg/mL (*p*<0.05) on HepG2 cells comparing to H-Total extract, H-H_2_O and H-*n*-BuOH fractions (Fig 1A). Six components from H-EtOAc fraction were isolated by column chromatography on silica gel. The SRB assays results indicated (Fig 1B) that DMQ and quercetin showed higher inhibitory effects (*p*<0.001) comparing to the geniposidic acid group (its cytotoxic activity was lower than other compounds), followed by 6,7-dihydroxycoumarin (*p* < 0.01) and rutin (*p* < 0.5), while methyl hexadecanoate were almost nontoxic to HepG2 cells (*p*>0.5). Between DMQ and quercetin, DMQ displayed much higher selectivity than quercetin (*p*<0.01), and its rates up to 9.4 ± 4.5, 2.8 ± 0.5 and 2.6 ± 0.4 folds at 125, 250 and 500 μmol/L, respectively (Fig 1C). So the H-EtOAc fraction and DMQ were selected to discuss the underlying anticancer mechanism. The *in vitro* cytotoxic activities of H-EtOAc fraction against six human cancer cell lines including HepG2, EC, SiHa, MCF-7, SGC-7901 and A549 were observed by the SRB assay as shown in Table 2. The results suggested that the cytotoxic activity on HepG2 cells was similar to EC cells (*p*>0.05) or significantly higher than those on other additional cell lines (*p*<0.01 or *p*<0.001). The dosage of H-EtOAc fraction of herb shown to be active (*p* < 0.5) against human Hep G2 cells were 62.5 μg/mL (its inhibition rate was higher than 32% than the control), and the amount of DMQ shown to be active toward Hep G2 cells was slightly lower than H-EtOAc fraction trentment for 24 h by the SRB assay, it was approximately 12.7μg/mL (which converted to molar concentration was about 50 μmol/L according to the molecular weight of DMQ is 254.2). The dose at which DMQ shown cytotoxic activity is a little high. Nevertheless, it displayed significant selectivity up to 9.4 fold to cancer cells and normal cells at 32 μg/mL (125μmol/L) (Fig 1C). In other words, DMQ may exhibit the higher cytotoxic activity for cancer cells and the relatively lower toxicity to normal cells. Hence, we selected it to investigate the mechanisms of cell-cycle distribution and apoptosis effects owing to its probability of acting as a promising lead compound for the further structure optimization. Moreover, considering the limited space of article, only HepG2 cells were selected to study the mechanisms of cell-cycle distribution and apoptosis effects.

**Table 2:** *In vitro* antitumor activity of H-EtOAc fraction against six human tumors ($\bar{x}$±SD).

Cell lines	Inhibition (%)			
	62.5 (μg/mL)	125 (μg/mL)	250 (μg/mL)	500 (μg/mL)
Hep G2	32.9 ± 4.6	32.8 ± 3.2	56.8 ± 5.1	71.2 ± 7.5
SGC-7901	3.7 ± 1.0***	5.8 ± 0.9***	10.7 ± 1.3***	17.5 ± 2.3***
SiHa	0.5 ± 0.1***	16.0 ± 1.2***	23.4 ± 2.5***	53.2 ± 4.1***
MCF-7	7.9 ± 1.8***	10.9 ± 1.9***	15.3 ± 2.2***	45.0 ± 3.4***
EC	25.7 ± 2.1**	35.9 ± 3.8	53.0 ± 4.2	68.8 ± 5.5
A549	9.6 ± 1.7***	15.0 ± 1.6***	17.2 ± 2.3***	38.3 ± 3.8***

Note: The SD values were obtained in six independent experiments (n = 6).

*p<0.05,

**p<0.01,

***p<0.001 versus Hep G2 cells.

*p<0.05 was considered significant.

### H-EtOAc Fraction and DMQ Induce Cells Damages and Cell Apoptosis Morphologically

**Fig 2:**
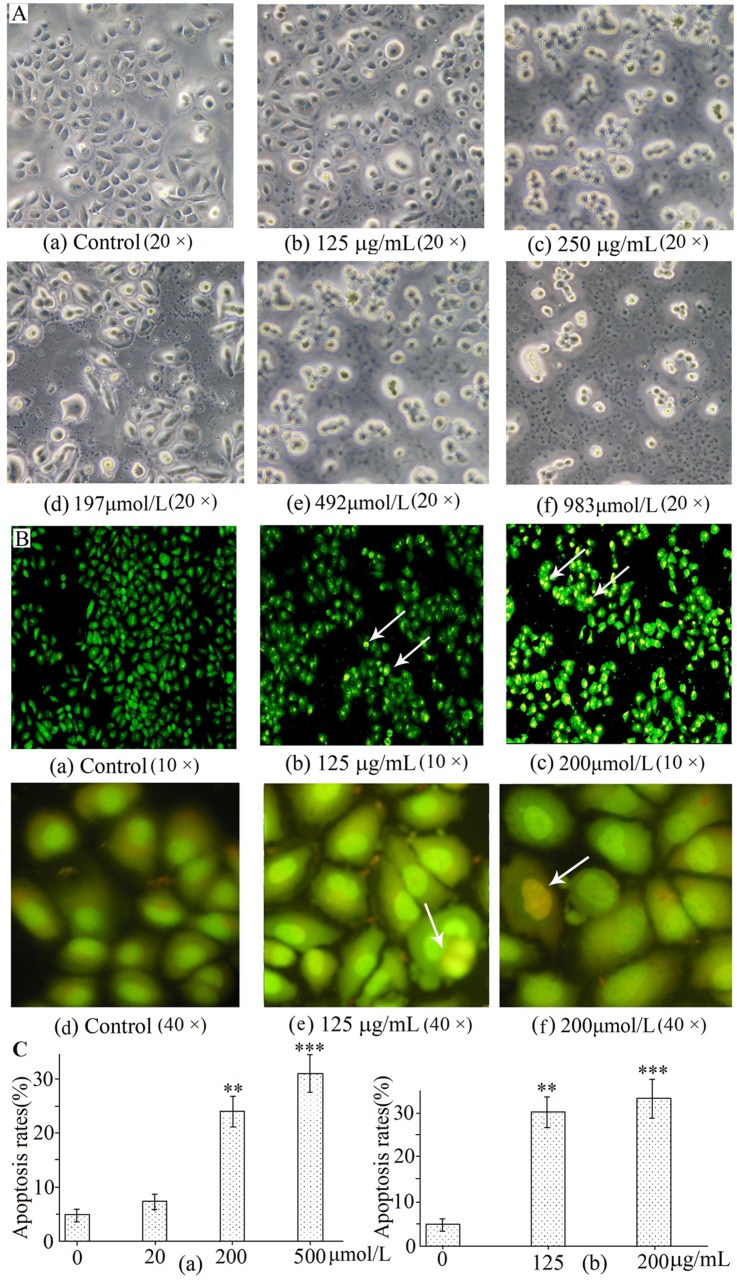
The Morphology Changes and apoptosis rates of H-EtOAc fraction and DMQ treated HepG2 cells. A(a)-(c). The morphology changes of HepG2 cells treated with gradient H-EtOAc fraction for 24 h using a visible light under light microscope for 24 h. A (d)-(f). The morphology changes of HepG2 cells treated with DMQ for 24 h. A visible light was used under light microscope (20 × magnification). B(a)-(c). Morphological observation of fluorescence microscope (10 × magnification) for 24 h. B(d)-(f). Morphological observation of fluorescence microscope (40 × magnification) for 24 h. A green excitation wavelength (460—495nm) and emission wavelength 510nm filter was used by staining with AO/EB. C(a). Apoptosis was measured on HepG2 cells treated with gradient DMQ for 24 h. C(b). Apoptosis was measured on HepG2 cells treated with H-EtOAc fraction for 24 h. Values are means ± SD of three independent experiments. * (*p*<0.05), ** (*p*<0.01) and *** (*p*<0.001) represented significant differences compared to the control.

Obvious damages (Fig 2A), such as cell rounding, shrinkage and loose arrangement, were observed under light microscope (20 × magnification) when HepG2 cells were treated with gradient H-EtOAc fraction (0, 125 and 250 μg/mL) and DMQ (197, 492 and 983 μmol/L) for 24 h, respectively. HepG2 cells were damaged in a dose dependent manner. The microscopic examination showed that live HepG2 cells in the control group displayed normal green nuclei, while H-EtOAc fraction and DMQ-treated cells showed membrane blebbing and bright dense granular masses of yellow or orange condensed or fragmented chromatin aggregated along the periphery of the nuclear membrane, indicating apoptosis (Fig 2B). Apoptotic rate was calculated as the percentage of apoptotic cells from at least 600 counted cells within the cells population with AO/EB staining as shown in Fig 2C. H-EtOAc fraction (0, 125 and 250 μg/mL) and DMQ (20, 200 and 500 μmol/L) demonstrated their anticancer effects on HepG2 cells through induction of apoptosis observed by AO/EB staining in a dose dependent manner (Fig 2C), as seen by the yellow-green apoptotic cells (Fig 2B) under fluorescence microscope (10 and 40 × magnification).

### The Apoptosis was Confirmed on HepG2 cells Treated with DMQ and H-EtOAc Fraction

**Fig 3:**
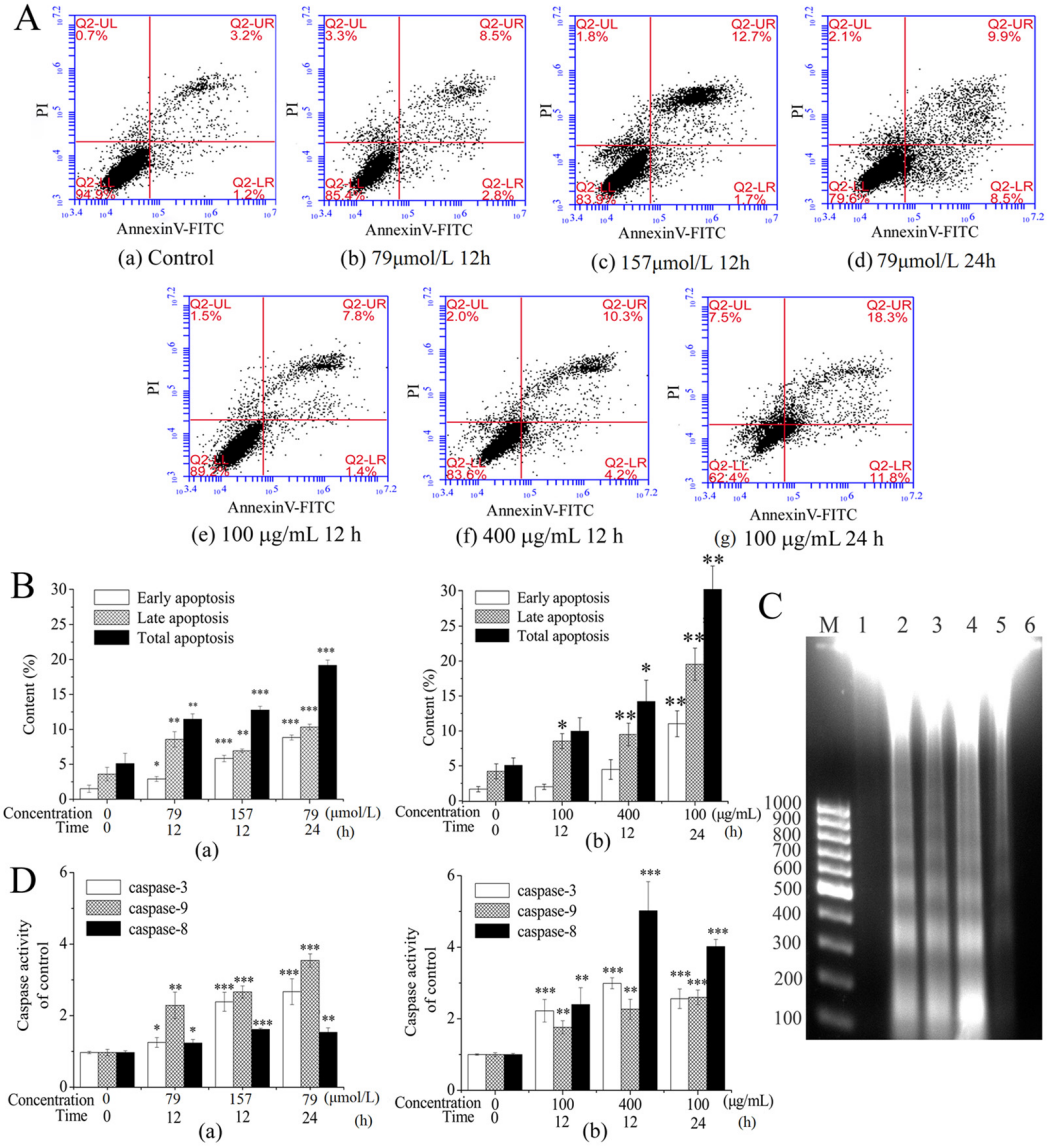
Apoptosis Induced by DMQ and H-EtOAc fraction of HepG2 Cells. A. Apoptosis was measured on HepG2 cells treated with gradient DMQ (a)-(d) and H-EtOAc fraction (e)-(g) by flow cytometry. B. The apoptosis rates were calculated of DMQ (a) and H-EtOAc fraction (b) on HepG2 cells. C. DNA ladder. Lane 1: Control; Lane 2–4: 79 μmol/L at 24 h, 157 μmol/L at 12 h and 157 μmol/L at 24 h for DMQ; Lane 5: 400 μg/mL at 24 h for H-EtOAc fraction; Lane 6: Negative group. D. The activities of caspase-3, -8, -9 proteases of HepG2 cells treated with DMQ (a) and H-EtOAc fraction (b). Values are means ± SD of three independent experiments. * (*p*<0.05), ** (*p*<0.01) and *** (*p*<0.001) represented significant differences compared to the control.

DMQ and H-EtOAc fraction induced apoptosis of HepG2 cells in a dose and time dependent manner, as the apoptosis was detected by annexin V-FITC/PI staining flow cytometry (Fig 3A and 3B). Different quadrants represent living cells (lower left quadrant), early apoptotic cells (lower right quadrant), late apoptotic cells (upper right quadrant) and mechanical damaged cells (upper left quadrant), respectively. The total apoptosis were the sum amounts of early apoptosis and late apoptosis. The total apoptosis rates of HepG2 cells were higher for DMQ treatment (Fig 3B(a)) and H-EtOAc fraction treatment (Fig 3B(b)) compared to the control. The apoptosis rates determined by AO/EB staining (Fig 2C) also increased in a dose dependent manner in H-EtOAc fraction and DMQ-treated cells which similar to flow cytometry method shown in Fig 3B(a) and 3B(b). Besides, DNA ladder confirmed the apoptosis clearly (Fig 3C) when HepG2 cells were treated with DMQ and H-EtOAc fraction. Furthermore, the dose and time dependent up-regulated caspase-3, -9 and -8 proteases activities (*p*<0.001) indicated the possible apoptosis mechanisms, including mitochondrial apoptotic and death receptor pathway (Fig 3D(a) and 3D(b)).

### Apoptosis Induced by DMQ and H-EtOAc Fraction on HepG2 cells through Activation of the Mitochondrial Apoptotic Pathway

**Fig 4:**
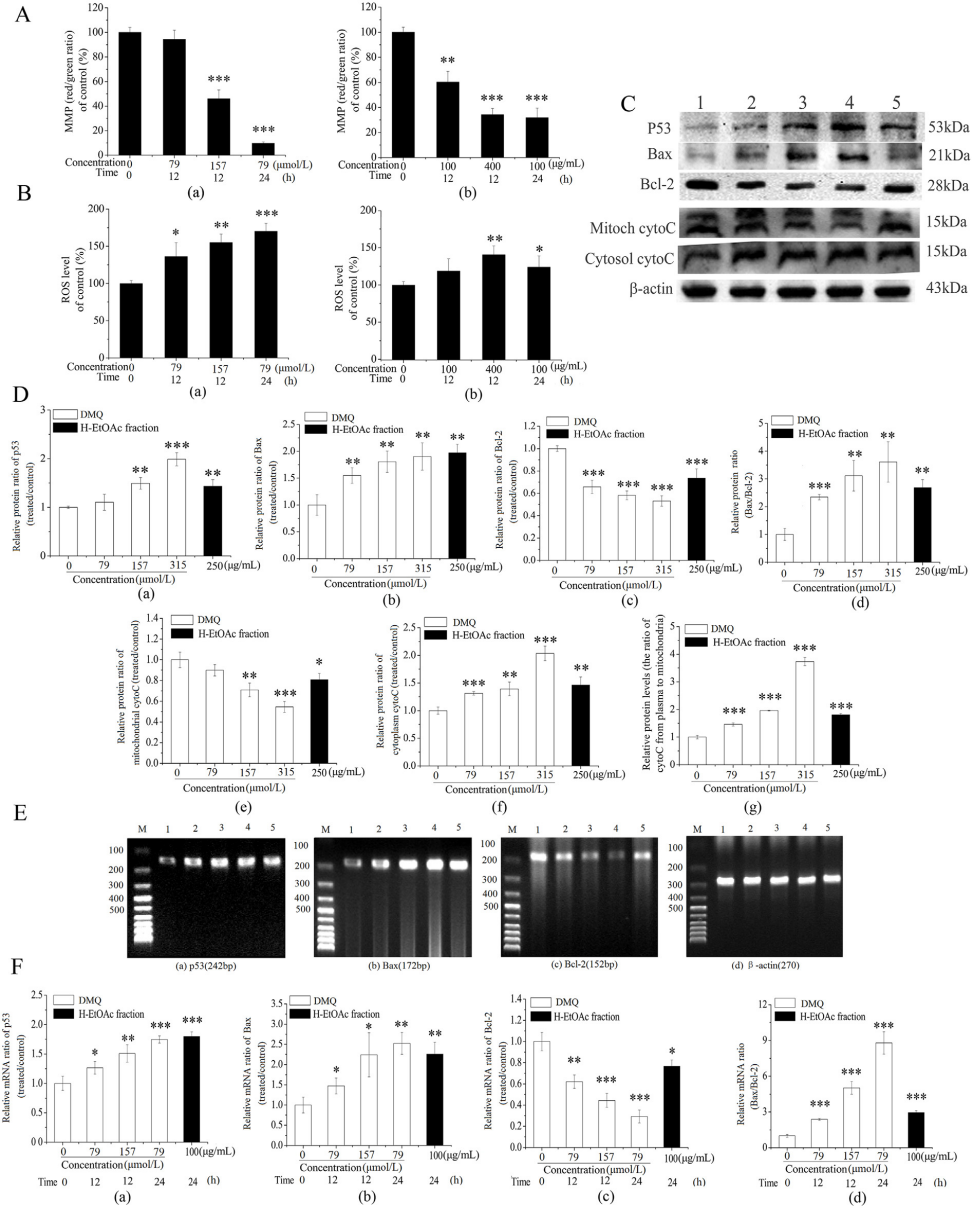
Mitochondrial Apoptotic Pathway Related to DMQ and H-EtOAc Fraction Treated HepG2 Cells. A. The MMP changes of HepG2 cells treated with DMQ (a) and H-EtOAc fraction (b). B. The production changes of intracellular ROS in HepG2 cells treated with DMQ (a) and H-EtOAc fraction (b). C. Western blot analysis of proteins expression levels of p53, Bax, Bcl-2, mitochondrial cyto C, cytoplasmic cyto C and β-actin. Lane 1: Control; Lane 2–4: 79, 157 and 315 μmol/L of DMQ for 24 h, respectively; Lane 5: 250 μg/mL of H-EtOAc fraction for 24 h. D. The protein expression levels of p53 (a) Bax (b) Bcl-2 (c) the ratio of Bax/Bcl-2 (d) mitochondrial cyto C (e) cytoplasmic cyto C (f) and the ratio of cytoplasmic cytoC/mitochondrial cyto C (g). The protein expression strength were analyzed by scanning densitometry using a Microtek ScanMaker 8700 (Zhongjing Inc. China) with ScanWizard 5 software. E. RT-PCR results of p53, Bax, Bcl-2 and β-actin. Lane 1: Control; Lane 2–4: 79 μmol/mL for 12 h, 157 μ/L for 12 h and 157 μmol/L for 24 h of DMQ, respectively; Lane 5: 100 μg/mL for 24 h of H-EtOAc fraction. F. The calculated mRNA expression levels of p53 (a) Bax (b) Bcl-2 (c) and the ratio of Bax/Bcl-2 (d). The mRNA expression strength were analyzed by scanning densitometry using a Microtek ScanMaker 8700 (Zhongjing Inc. China) with ScanWizard 5 software. Values are means ± SD of three independent experiments. * (*p*<0.05), ** (*p*<0.01) and *** (*p*<0.001) represented significant differences compared to the control.

Significant reduction of MMP (Fig 4A) and increased intracellular ROS production (Fig 4B) were observed on DMQ and H-EtOAc fraction treated HepG2 cells in a dose and time dependent manner by flow cytometry. MMP (Fig 4A(a)) was decreased (*p*<0.001) and ROS (Fig 4B(a)) was increased (*p*<0.01) in HepG2 cells treated with DMQ compared to the control. In addition, MMP (Fig 4A(b)) were declined (*p*<0.001) and ROS (Fig 4B(b)) were raised (*p*<0.05) on H-EtOAc fraction treated HepG2 cells compared to the control, respectively. To confirm whether mitochondrial apoptotic pathway was involved directly, the expressions of p53, Bax and Bcl-2 were detected by western blot assays (Fig 4C and 4D). Treatment with DMQ resulted in the up-regulation of Bax (Fig 4D(b)), down-regulation of Bcl-2 (Fig 4D(c)) on HepG2 cells in a dose dependent manner (*p*<0.01). And treatment with H-EtOAc fraction (250μg/ml) also resulted in the up-regulation of Bax (*p*<0.01), down-regulation of Bcl-2 (*p*<0.001) on HepG2 cells (Fig 4D(b) and 4D(c)) compared to the control. The ratio of Bax/Bcl-2 was increased (*p*<0.01) with DMQ treatment and H-EtOAc fraction treatment for 24 h compared to the control, respectively (Fig 4D(d)). The increase of Bax/Bcl-2 ratio, which was advanced by increased expression of p53 (*p*<0.01) (Fig 4D(a)), could promote the collapse of the high MMP (Fig 4A), increase of ROS (Fig 4B), eventually leading to the opening of mitochondrial outer membrane permeabilization transition (PT) pore. In addition, released cyto C from mitochondria to cytoplasm was checked. Cyto C from mitochondria decreased significantly (*p*<0.01) (Fig 4C and 4D(e)) and cyto C of cytoplasm increased remarkably (*p*<0.01) (Fig 4C and 4D(f)) compared to that of the control by western blot assays. The ratio of cytoplasmic cytoC/mitochondrial cytoC was up-regulated by DMQ and H-EtOAc fraction trentment compared to the control (*p*<0.001) respectively (Fig 4D(g)), it can be concluded that DMQ and H-EtOAc fraction could promote the release of cyto C from mitochondria to cytoplasm in a dose dependent manner, which could activate caspase-9 and caspase-3 (Fig 3D) to cleave the target protein to execute cell apoptosis. Therefore, mitochondrial apoptotic pathway was involved in HepG2 cells treated with DMQ and H-EtOAc fraction. Furthermore, the mRNA expressions of p53, Bax (Fig 4E, S3 and S1 Figs, Fig 4F(a) and 4F(b)) were raised remarkably (*p*<0.01), while that of Bcl-2 (Fig 4E, S2 Fig and Fig 4F(c)) was reduced significantly (*p*<0.01). The ratio of Bax/Bcl-2 (Fig 4F(d)) was increased for DMQ treatment and H-EtOAc fraction treatment in HepG2 cells compared to the control (*p*<0.001), respectively, in dose and time dependent manner, which consistent with the western blot results.

### Apoptosis Induced by DMQ and H-EtOAc Fraction on HepG2 cells through Activation of the Death Receptor Pathway

**Fig 5:**
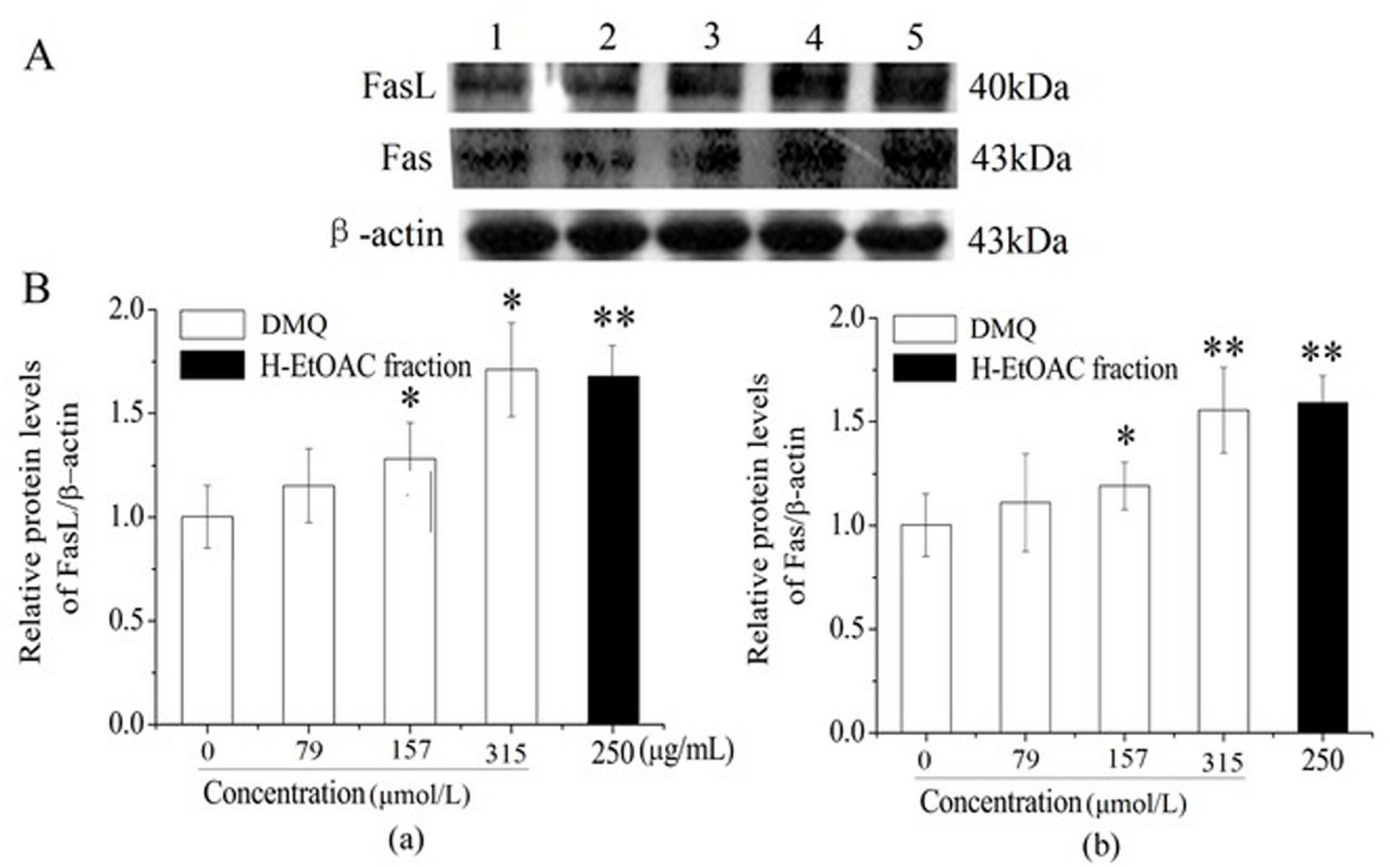
Death Receptor Pathway Related to HepG2 Cells Treated with DMQ and H-EtOAc Fraction. A. Western blot analysis of FasL and Fas. Lane 1: Control; Lane 2–4: 79, 157 and 315 μmol/L of DMQ for 24 h, respectively; Lane 5: 250 μg/mL of H-EtOAc fraction for 24 h. B. The protein expression levels of FasL (a) and Fas (b). Values are means ± SD of three independent experiments. * (*p*<0.05), ** (*p*<0.01) and *** (*p*<0.001) represented significant differences compared to the control.

To detect whether death receptor pathway was involved clearly in apoptosis after gradient DMQ and H-EtOAc fraction treatment on HepG2 cells for 24 h, the caspase-3 and -8 proteases activities (Fig 3D), and the contents changes of three proteins which related to death receptor pathway were investigated by western blot and PT-PCR assays, including p53 (Fig 4C, 4E, 4D(a) and 4F(a)), Fas and FasL (Fig 5A and 5B). From the heightened expression (*p*<0.01) of caspase-3, -8, FasL, Fas, and p53 which were stimulated by DMQ, H-EtOAc fraction, we could infer the activation of FasL with Fas, further activation of caspase-8 and caspase-3 (Fig 3D), leading to cell apoptosis.

### Apoptosis Induced by DMQ and H-EtOAc Fraction was Dependent on both Caspase-8 and Caspase-9

**Fig 6:**
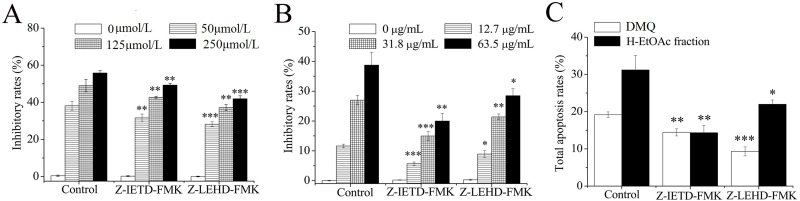
Caspase-8 and Caspase-9 Dependent Apoptosis of HepG2 Cells Induced by DMQ and H-EtOAc Fraction. A. HepG2 cells were incubated with caspase-8 inhibitor (Z-IETD-FMK: 20 μmol/L) or caspase-9 inhibitor (Z-LEHD-FMK: 20 μmol/L) for 30 min followed by DMQ at 0, 50, 125 and 250 μmol/L for 24 h, the inhibitory rates were detected by SRB assays. B. HepG2 cells were incubated with Z-IETD-FMK or Z-LEHD-FMK for 30 min and subsequently treated with H-EtOAc fraction at 0, 12.7, 31.8 and 63.5 μg/mL for 24 h, and the inhibitory rates were determined by SRB assays. C. HepG2 cells were pre-incubated with Z-IETD-FMK or Z-LEHD-FMK for 30 min before treated with DMQ (79 μmol/L) or H-EtOAc fraction (100 μg/mL) for 24 h, the cellular apoptotic rates were measured by flow cytometry. Values are means ± SD of three independent experiments. * (*p*<0.05), ** (*p*<0.01) and *** (*p*<0.001) represented significant differences compared to the control.

As indicated, compared to the control, the H-EtOAc fraction (100 μg/mL 12 h, 400 μg/mL 12 h, 100 μg/mL 24 h) treatment did not induce much caspase-9 activation (Fig 3D(b)), nor the cytoC release in HepG2 cells at the measured concentrations (250 μg/mL 24 h) (Fig 4C and 4D(e)–4D(g)). In contrast, the activation of caspase-8 was dominant (Fig 3D(b)). Meanwhile, DMQ (79 μmol/L 12 h, 157 μmol/L 12 h, 79 μmol/L 24 h) induced a much more stronger activation of caspase-9 than caspase-8 activation (Fig 3D(a)), the cyto C release at 157 and 315 μmol/L was significant (Fig 4C and 4D(e)–4D(g)). Furthermore, to detect whether the apoptosis was dependent on caspase-8, caspase-9 or both of them, HepG2 cells were incubated in present or absent of caspase-8 inhibitor (Z-IETD-FMK) or caspase-9 inhibitor (Z-LEHD-FMK) before DMQ or H-EtOAc fraction treatment. As seen from Fig 6, when HepG2 cells were pre-treated with Z-IETD-FMK or Z-LEHD-FMK, the inhibitory and apoptosis rates of DMQ and H-EtOAc fraction treatment decreased significantly (*p*<0.01). Moreover, the inhibitory and apoptosis rates of HepG2 cells pre-treated with Z-LEHD-FMK and treated with DMQ were down-regulated more than those with Z-IETD-FMK pre-treatment (Fig 6A and 6C). Meanwhile, pre-treated with Z-IETD-FMK on HepG2 cells, the inhibitory rates of the H-EtOAc fraction treatment were down-regulated more than Z-LEHD-FMK pre-treatment, which consistent with the results of apoptosis rates (Fig 6B and 6C). Therefore, mitochondrial apoptotic pathway and death receptor pathway were both involved in apoptosis of HepG2 cells, which was detected to be dependent on caspase-8 and caspase-9, induced by DMQ and the H-EtOAc fraction (Figs 4–6). Moreover, DMQ was mainly through mitochondrial apoptotic pathway, and the H-EtOAc fraction mainly through death receptor pathway.

### P53 abrogation reduces the anticancer effects of DMQ and H-EtOAc fraction

Literature reports suggest that p53 plays a key role in the regulation of apoptosis via decreasing the expression of the apoptosis-suppressing gene Bcl-2, meanwhile increasing the expression of Bax that encodes a dominant inhibitor of the Bcl-2 protein [18]. To determine whether tumor suppression factor p53 and its downstream molecule, cyclin-dependent kinase inhibitor p21, are directly involved in the antiproliferative effect of DMQ and H-EtOAc fraction, HepG2 cells treated with inhibitor of p53, PFT-α at 20 μmol/L for 24 h did not exhibit significant cytotoxicity (*p* > 0.05) as evaluated by SRB Assay (Fig 1D). When cells were pretreated with DMQ (Lane 4, 50μmol/L for 24 h) and H-EtOAc fraction (Lane 5, 100 μg/ml for 24 h) followed by PFT-α (20 μmol/L) treatment, cell viability did not significantly increased (*p* > 0.05) than the group treated with 20 μmol/L PFT-α without DMQ and H-EtOAc fraction (Lane 1) treatment by SRB Assay (Fig 1E). Furthermore, the mRNA (Fig 4E(a), S3 Fig and Fig 4F(a)) and protein (Fig 4C and 4D(a)) expressions of p53 in HepG2 cells treated with DMQ (50μmol/L for 24 h) and H-EtOAc fraction (100 μg/ml for 24 h) were raised remarkably (*p*<0.01) without PFT-α treatment, however, in the presence of PFT-α (inhibitor of p53, 20μmol/L), the mRNA level of p53 and its downstream molecule, cyclin-dependent kinase inhibitor p21 (Fig 1F and 1G), were no longer significantly increased (*p* > 0.05). Based on our results obtained in mitochondrial apoptosis pathway, we presumed that induction of p53 may be inhibited by its specific inhibitor PFT-α, which reversibly block p53-dependent signaling/function transcriptional activation and apoptosis (Fig 1E). To ascertain the specificity of p53, in regulation of FASR/FasL, HepG2 cells will be transfected with specific p53 siRNA in our collaborating laboratory.

### Cell cycle analysis was Detected in 1,3-dihydroxy-2-methylanthra- quinone and H-EtOAc Fraction Treated HepG2 Cells

**Fig 7:**
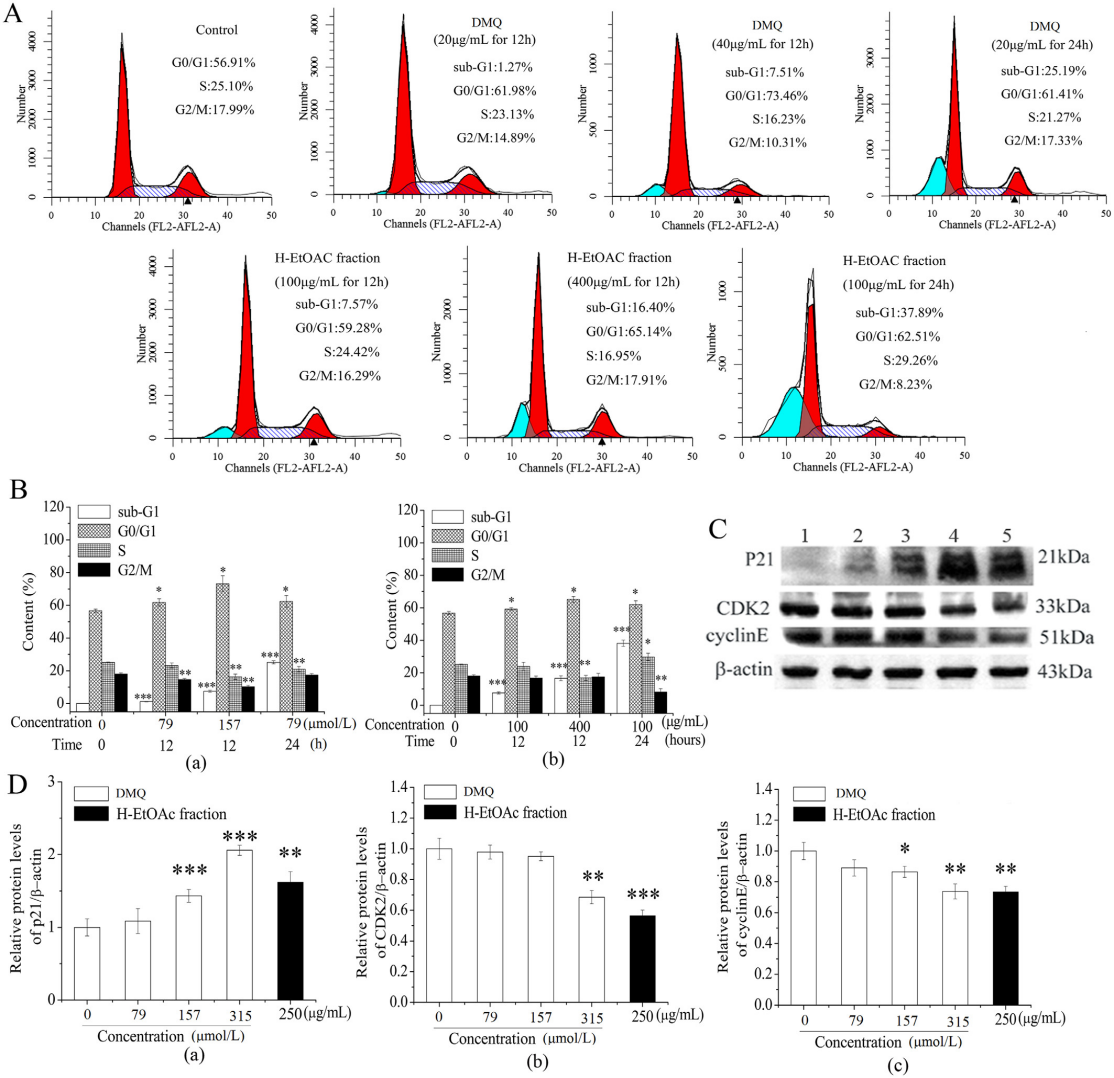
Cell Cycle Arrest of HepG2 Cells Treated with DMQ and H-EtOAc Fraction. A. The cell cycle distribution of treated HepG2 cells. B. Contents of each phase of HepG2 cells treated with DMQ (a), H-EtOAc fraction (b). C. Western blot analysis of p21, CDK 2 and cyclin E. Lane 1: Control; Lane 2–4: 79, 157 and 315 μmol/L of DMQ for 24 h, respectively; Lane 5: 250 μg/mL of H-EtOAc fraction for 24 h. D. The protein expression levels of p21 (a), CDK 2 (b) and cyclin E (c). The protein expression strength were analyzed by scanning densitometry using a Microtek ScanMaker 8700 (Zhongjing Inc. China) with ScanWizard 5 software. Values are means ± SD of three independent experiments. * (*p*<0.05), ** (*p*<0.01) and *** (*p*<0.001) represented significant differences compared to the control.

Cell cycle analysis of HepG2 cells treated with gradient DMQ and H-EtOAc fraction, were determined by flow cytometry assays (Fig 7A and 7B). Treatments with DMQ and H-EtOAc resulted in slightly lower proportions of cells in S-phase and G2/M, which may suggest that cycling cells are more sensitive to the tested extracts. In addition, there is no clear time-dependent effect on the growth of HepG2 cells treated with DMQ and H-EtOAc fraction between 12 h and 24h by blocking cell cycle at G0/G1 stage in Fig 7B, and experimental time (only 12 versus 24h) was too short in view of the doubling time of 50 to 60h (http://bioinformatics.hsanmartino.it/hypercldb/cl1644.html). Besides, a gate had been used for selecting smaller apoptotic cell population (neither cellular debris, also nor clustered cells) than normal cells in FSC × SSC scatter plots, and the sub-G1 phase peaks (blue peaks) of the histogram in Fig 7A were presented as the apoptotic cell populations which distinguished from a large number of cells. From Fig 7A, we could find that sub-G1 peak was a typical characteristic feature of apoptosis, which increased significantly (*p*<0.001) compared to the control. It was concluded that DMQ and H-EtOAc fraction could induce apoptosis in dose and time dependent manner (Fig 7A and 7B). In order to further confirm if HepG2 cells treated with DMQ and H-EtOAc fraction were inhibited growth by blocking cell cycle at G0/G1 stage, contents of proteins, such as cyclin dependent kinase cyclin E, CDK 2, and p21 Kip, a cyclin kinase inhibitors (CKI) protein, antagonizing the binding of cyclinE with CDK 2 [19], were detected by western blot assays (Fig 7C and 7D). After HepG2 cells were treated with gradient DMQ and H-EtOAc fraction respectively, the expression of cyclin E (Fig 7C and 7D(c)) and CDK 2 (Fig 7C and 7D(b)) were decreased and p21 (Fig 7C and 7D(a)) was increased (*p*<0.01).

## Discussion

The H-EtOAc fraction and its main component DMQ showed significantly anticancer effects on HepG2 cells (Fig 1A and 1B). In addition, DMQ showed high selectivity rates between HepG2 and HL-7702 cells (Fig 1C). DMQ and H-EtOAc fraction inhibited cell growth by inducing apoptosis, which was confirmed using AO/EB staining (Fig 2B), flow cytometry (Fig 3A and 3B), and DNA ladder assays (Fig 3C) *in vitro*. In addition, the increased caspase-3, 9 (mitochondrial apoptotic pathway), and caspase-8 (death receptor pathway) proteases activities (Fig 3D) indicated possible apoptotic mechanisms. HepG2 cells were pre-treated with Z-IETD-FMK, Z-LEHD-FMK or none, and then co-treated with DMQ or H-EtOAc fraction, the inhibitory and apoptosis rates results indicated that the apoptosis induced by DMQ or H-EtOAc fraction was dependent on both caspase-8 and caspase-9 (Fig 6). Apoptosis induced by the compound DMQ was mainly through mitochondrial apoptotic pathway, and H-EtOAc fraction was mainly through death receptor pathway. The pro-apoptotic transcription factor p53, targets multiple proteins (Fas, Bax, Bcl-2, and p21) to participate in mitochondrial apoptotic and death receptor pathways as well as cell cycle arrest [20].

H-EtOAc fraction and DMQ could induce apoptosis of HepG2 cells, which promoted the up-regulation of p53 (Fig 4C, 4E, 4D(a) and 4F(a)). Mutations in tumor protein p53 (TP53) are very frequent in cancer (50% of all tumor) and resistance to primary medical therapy, especially in breast cancer. TP53 protein acts as a tumor suppressor, which means that it is essential for regulating cell division by keeping cells from growing and dividing too fast or in an uncontrolled way. Earlier studies revealed that overexpression of p53, the protein coded for by the TP53 gene, is a prognostic factor [21]. In this paper, experimental data suggested a key role for p53 in apoptosis in response to H-EtOAc fraction and DMQ trentment. The mRNA (Fig 4E(a), S3 Fig and Fig 4F(a)) and protein (Fig 4C and 4D(a)) expressions of p53 in HepG2 cells treated with DMQ and H-EtOAc fraction were raised remarkably (*p* <0.01), but induction of p53 (no overexpression of p53) may be inhibited by its specific inhibitor PFT-α, which reversibly block p53-dependent signaling/function transcriptional activation and apoptosis (Fig 1E). Considering that DMQ and H-EtOAc fraction induce cell apoptosis via p53, tumors with inactivating mutations in TP53 may be resistant to these extracts. Investigation is warranted to address whether tumors with TP53 mutations are more resistant to treatment with Hedyotis Diffusa Willd.

P53 also exerted its proapoptotic function by increasing the ratio of Bax/Bcl-2 thereby activating the mitochondrial apoptotic pathway. The ratio of Bax/Bcl-2 increased (Fig 4C, 4E and 4D(b)–4D(d), S1, S2 Figs and Fig 4F(b)–4F(d)), leading to a fall in the high MMP level (Fig 4A) and the increase in ROS as shown by flow cytometry (Fig 4B), eventually resulting in the opening of the mitochondrial outer membrane PT pores. Therefore, intermembrane proteins were released into the cytoplasm including cyto C, which plays a crucial role in the signal transduction cascade leading to cell apoptosis [22–24]. The release of cyto C from the mitochondria to the cytoplasm was examined using western blot analysis (Fig 4C and 4D(e)–4D(g)). The conjugation of cytoC with the apoptosis protease activating factor-1 (APAF-1) in the presence of ATP exposed its caspase recruitment domains (CARD) and induced the aggregation of procaspase-9 to form the death-inducing signaling complex (DISC) [25]. After undergoing conformational changes, procaspase-9 was cleaved and activated, and the released caspase-9 activated the downstream caspase-3 (Fig 3D) leading to cell apoptosis.

The death receptor pathway was triggered by up-regulated extracellular death ligand protein FasL and intracellular death receptor protein Fas. In HepG2 cells, DMQ and the H-EtOAc fraction induced the up-regulation of p53 (Fig 4C, 4E, 4D(a) and 4F(a)), which further promoted the activation of FasL and Fas to activate the death receptor pathway (Fig 5A and 5B). The ligation of Fas by the up-regulated FasL promoted their clustering to form a complex. This action, recruited the adaptor protein Fas-associated protein with death domain (FADD) through death domain interactions with proteins such as procaspase-8, following the activation of caspase-3 (Fig 3D), leading to cell death [26].

As shown in Fig 4C, 4E, 4D(a) and 4F(a), p53 induced p21 expression and enhanced its inhibition of cell cycle and growth. Flow cytometry revealed that the HepG2 cells treated with DMQ and H-EtOAc fraction could induce apoptosis in dose and time dependent manner (Fig 7A). CyclinE is common in cyclins, which is necessary for CDK 2 ligation and activation. The CDK 2 is a serine/threonine protein kinase, which has significant sequence homology with cyclinE protein, which facilitates their ligation. The activated p21 Kip, a CKI protein, antagonized the binding of cyclinE with CDK 2 thereby blocking the cell cycle in the G0/G1 phase [27]. Once CDK 2 binds appropriately to cyclin E, the favored conformational changes activate it and catalyze the substrate proteins to prompt normal cell cycle circulations. Activated p21, antagonizes the activity of the cyclinE and CDK 2 in the late G0/G1 stage, preventing the transition to the next stage [28]. Treatment with DMQ and H-EtOAc fraction could enhance the expression of p21 (Fig 7C and 7D(a)). However, if HepG2 cells treated with DMQ and H-EtOAc fraction were inhibited by blocking cell cycle, still need to be further confirmed through BrdU incorporation and p-Rb assays considering the small difference seen in cyclin-dependent kinase CDK 2 and cyclin E.

## Conclusions

In conclusion, our findings suggested that DMQ and the H-EtOAc fraction from HDW could be valuable for their inhibitory effects against HepG2 carcinoma cells. This inhibition may involve caspase-8 and caspase-9 dependent apoptosis, which was mediated *via* the mitochondrial apoptotic and death receptor pathways. However, the *in vivo* therapeutic effects are still unknown and require further investigation.

## Supporting Information

S1 Fig Raw supplementary RT-PCR figure of bax in Fig 4E. (TIF)

S2 Fig Raw supplementary RT-PCR figure of bcl-2 in Fig 4E. (TIF)

S3 Fig Raw supplementary RT-PCR figure of p53 in Fig 4E. (TIF)

S4 Fig Raw supplementary RT-PCR figure of beta in Fig 4E. (TIF)

## Abbreviations

HDW: *Hedyotis diffusa* Willd
FBS: Fetal bovine serum
BCA: Bicinchoninic acid assay
ECL: Enhanced chemiluminescence
SRB: Sulforhodamine B assay
TCA: Trichloroacetic acid
ATP: Adenosine triphosphate
UPLC-MS: Ultraperformance liquid chromatography and mass spectrometry method
cytoC: cytochromeC
AO and EB: Acridine orange and ethidium bromide assay
PS: Phosphatidylserine
FITC: Fluorescein isothiocyanate
PI: Propidine iodide
JC-1: 5,5',6,6'-tetrachloro-1,1',3,3'-tetrethyl benzimidazoly carbocyanine iodide dye
MMP: Mitochondrial membrane potential
DCFH-DA: 20,70-dihydrofluorescein-diacetate
DCFH: 20,70-dihydrofluorescein
DCF: Dichlorofluorescein
ROS: Reactive oxygen species
PMSF: Phenylmethanesulfonyl fluoride
SDS-PAGE: Sodium dodecyl sulphate-polyacrylamide gels
NC: Nitrocellulose
TBST: Tris-buffered saline plus Tween-20
RT-PCR: Reverse transcription-polymerase chain reaction
PFT-α: pifithrin-α
TP53: tumor protein p53
PT pore: Permeabilization transition pore
TNF: tumor necrosis factor
CKI: Cyclin kinase inhibitors
APAF-1: Apoptosis protease activating factor-1
CARD: Caspase recruitment domains
DISC: Death-inducing signaling complex
FADD: Fas associated death domain
