# Supplementary figures and images for: Anticancer Effects of 1,3-Dihydroxy-2-Methylanthraquinone and the Ethyl Acetate Fraction of Hedyotis Diffusa Willd against HepG2 Carcinoma Cells Mediated via Apoptosis
Source: PLoS One. 2016 Apr 11;11(4):e0151502. doi: 10.1371/journal.pone.0151502 (PMC4827846; doi:10.1371/journal.pone.0151502)

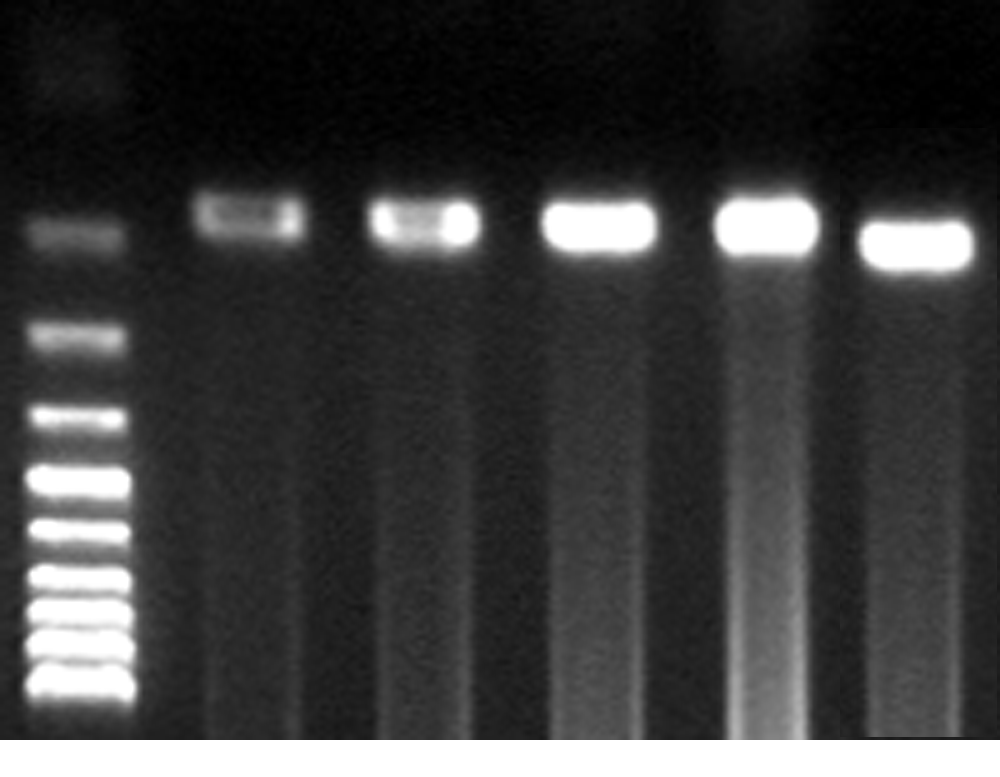

Supplement: S1 Fig — (TIF) [file pone.0151502.s001.tif]

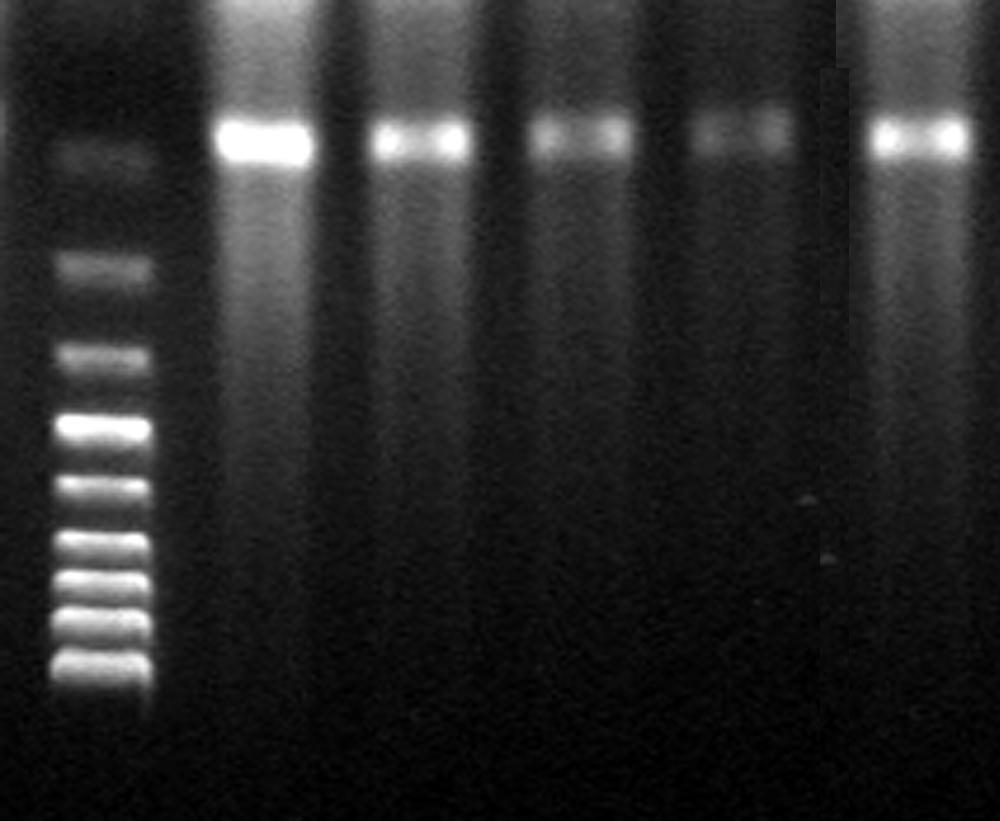

Supplement: S2 Fig — (TIF) [file pone.0151502.s002.tif]

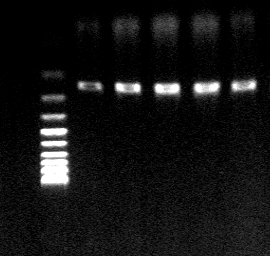

Supplement: S3 Fig — (TIF) [file pone.0151502.s003.tif]

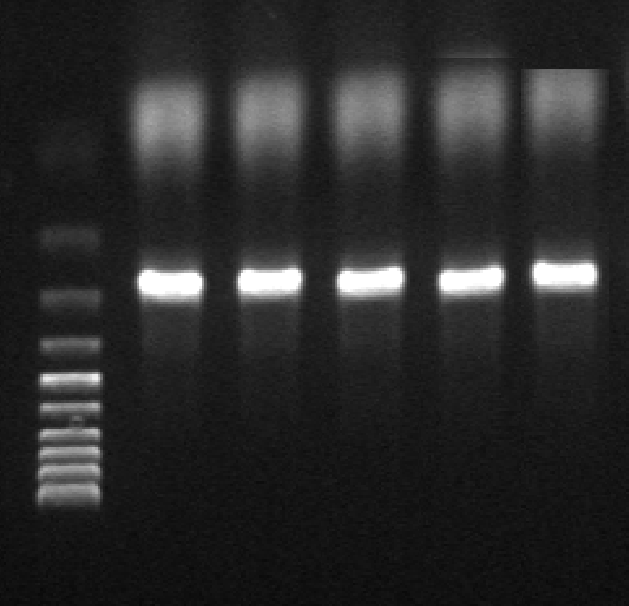

Supplement: S4 Fig — (TIF) [file pone.0151502.s004.tif]
